# Sequential Processing and the Matching-Stimulus Interval Effect in ERP Components: An Exploration of the Mechanism Using Multiple Regression

**DOI:** 10.3389/fnhum.2016.00339

**Published:** 2016-06-30

**Authors:** Genevieve Z. Steiner, Robert J. Barry, Craig J. Gonsalvez

**Affiliations:** ^1^Centre for Psychophysics, Psychophysiology, and Psychopharmacology, Brain and Behaviour Research Institute, and School of Psychology, University of WollongongWollongong, NSW, Australia; ^2^The National Institute of Complementary Medicine (NICM), Western Sydney UniversityPenrith, NSW, Australia; ^3^School of Social Sciences and Psychology, Western Sydney UniversityPenrith, NSW, Australia

**Keywords:** event-related potentials (ERPs), target-to-target interval (TTI), nontarget-to-nontarget interval (NNI), sequence effects, interstimulus interval (ISI), probability, oddball task, P300

## Abstract

In oddball tasks, increasing the time between stimuli within a particular condition (target-to-target interval, TTI; nontarget-to-nontarget interval, NNI) systematically enhances N1, P2, and P300 event-related potential (ERP) component amplitudes. This study examined the mechanism underpinning these effects in ERP components recorded from 28 adults who completed a conventional three-tone oddball task. Bivariate correlations, partial correlations and multiple regression explored component changes due to preceding ERP component amplitudes and intervals found within the stimulus series, rather than constraining the task with experimentally constructed intervals, which has been adequately explored in prior studies. Multiple regression showed that for targets, N1 and TTI predicted N2, TTI predicted P3a and P3b, and Processing Negativity (PN), P3b, and TTI predicted reaction time. For rare nontargets, P1 predicted N1, NNI predicted N2, and N1 predicted Slow Wave (SW). Findings show that the mechanism is operating on separate stages of stimulus-processing, suggestive of either increased activation within a number of stimulus-specific pathways, or very long component generator recovery cycles. These results demonstrate the extent to which matching-stimulus intervals influence ERP component amplitudes and behavior in a three-tone oddball task, and should be taken into account when designing similar studies.

## Introduction

### Background

Traditionally, the oddball task is used to study the effects of stimulus novelty and significance on information processing. Participants are required to identify, or respond to (e.g., count, button press) low probability target stimuli that are temporally dispersed in a background of frequent nontarget (standard) stimuli. Within oddball tasks, the stimulus-to-matching-stimulus interval is the time separating presentations of a particular stimulus category (e.g., target-to-target interval, TTI; nontarget-to-nontarget interval, NNI). Depending on the variant of the oddball task employed, the matching-stimulus interval can contain any combination of nonmatching stimuli or silence. Changing these intervals alters the temporal probability of the stimulus of interest, and the effect of these alterations has been explored in components of the event-related potential (ERP), particularly the P300.

The P300 component of the ERP is a positive deflection in the waveform with a parietal scalp distribution, occurring ~300 ms post-stimulus, and comprises several independent components (P3a, P3b, Novelty P3). Many early oddball ERP studies explored the effects of stimulus sequence (also referred to as local probability; Squires et al., 1976, 1977; Hermanutz et al., 1981; Johnson and Donchin, 1982; Sams et al., 1983, 1984; Verleger, 1987; Leuthold and Sommer, 1993; Starr et al., 1997), interstimulus interval (ISI; Fitzgerald and Picton, 1981; Polich, 1990a,b; Miltner et al., 1991), global probability (i.e., the overall proportion of stimuli in a particular category; Duncan-Johnson and Donchin, 1977; Polich et al., 1991; Polich and Bondurant, 1997), and temporal probability (i.e., the probability that a stimulus from a particular category will occur within a given time period; Fitzgerald and Picton, 1981) on the P300, reporting a relatively consistent pattern of results—decreases in global and temporal probability, and increases in sequence length and ISI all increase P300 amplitude. However, in a review of this previous research, Gonsalvez et al. (1999) argued that many of those early findings may be attributable to changes in the matching-stimulus interval, as manipulations of stimulus sequence, ISI, and global and temporal probability unavoidably alter the TTI and NNI.

Gonsalvez and colleagues then went on to demonstrate that P300 is strongly affected by TTI in a number of carefully designed studies (Gonsalvez et al., 1999, 2007; Gonsalvez and Polich, 2002; Croft et al., 2003; Steiner et al., 2013a,b, 2014a). It was shown that increases in TTI systematically enlarge target P300 amplitude, and reduce target P300 latency, while global probability, stimulus sequence, and ISI are held constant. In other words, TTI is an independent predictor of P300. All those studies manipulated TTIs using a range of ISIs within (e.g., Gonsalvez et al., 1999) or between (e.g., Gonsalvez and Polich, 2002) stimulus blocks, or by embedding fixed interval ranges (e.g., 1–15 s) within a stimulus sequence using a fixed-ISI (e.g., Steiner et al., 2013b).

A similar pattern of results has been demonstrated for the nontarget P300 in an equiprobable task (Steiner et al., 2014a), with increases in NNI enhancing nontarget P300 amplitude. However, NNI effects seem to be contingent on the task-relevance assigned to nontarget stimuli (Sawaki and Katayama, 2006), with other studies not demonstrating NNI effects in low probability nontarget P300 amplitude (Steiner et al., 2013b), or changes in functional magnetic resonance imaging (fMRI) bold signal (Stevens et al., 2005).

### Effects in non-P300 ERP components

Some of the early ERP studies mentioned above also explored changes in a range of ERP components, and reported findings similar to those found in the P300 for manipulations of matching-stimulus interval. For example, when the number of stimulus repetitions preceding a deviant (a target or a rare nontarget) increases, deviant component magnitude increases occur for N1 (Hermanutz et al., 1981; Verleger, 1987; Starr et al., 1997), mismatch negativity (MMN; Sams et al., 1983; Imada et al., 1993; Haenschel et al., 2005) and N2 amplitude (Sams et al., 1983); when ISI is longer, N1 (Woods et al., 1980; Woods and Courchesne, 1986; Polich, 1990b; Miltner et al., 1991; Teder et al., 1993; Polich and Bondurant, 1997; Budd et al., 1998; Čeponienė et al., 1998; Coch et al., 2005) and P2 amplitudes increase (Woods and Courchesne, 1986; Polich, 1990b; Miltner et al., 1991); when the global probability of a stimulus decreases, MMN (Näätänen and Gaillard, 1983) and N2 amplitudes increase (Polich, 1990b); and when temporal probability decreases, MMN (Sabri and Campbell, 2001) and P2 (Fitzgerald and Picton, 1981) amplitudes increase. However, when compared to the highly consistent response profile reported for target P300, the pattern of results is significantly more variable for P1^1^ (Thomas et al., 2009), N1 (Kenemans et al., 1991; Polich and Bondurant, 1997; Thomas et al., 2009), MMN (Javitt et al., 1998), P2 (Polich, 1990b; Polich and Bondurant, 1997), and N2 (Hermanutz et al., 1981; Polich and Bondurant, 1997; Thomas et al., 2009), and this variability is largely contingent upon the characteristics of the paradigm employed (e.g., two- or three-stimulus oddball, Go/NoGo, etc.).

In a recent study, Steiner et al. (2014b) aimed to address a number of inconsistencies in the findings from probability, sequence, and ISI research by systematically exploring the effect of TTI and NNI in a range of ERP components. N1, P2, and P3b amplitudes were found to increase as TTI and NNI increased, but Processing Negativity (PN; a temporally-distributed negativity late in the N1-latency range; Näätänen et al., 1978), and the frontally-negative/parietally-positive Slow Wave (SW; Courchesne, 1983; Courchesne et al., 1984) did not show the same pattern of results. Steiner et al. (2014b) also explored the mechanism of matching-stimulus interval effects and its origin in sequential processing, and as non-sequential ERP components elicited similar response profiles, it was concluded that a similar temporal mechanism operating on different stages of stimulus processing was underpinning TTI and NNI effects in early (N1) and late (P300) ERP components.

### Aims and hypotheses

The current study aimed to expand on Steiner et al. (2014b) by further exploring the mechanism of interval effects in sequential processing, and determining whether the previous pattern of results could be obtained with a more commonly used paradigm. Hence, we aimed to increase the generalisability of results by using a conventional oddball task with no TTI/NNI constraints. As outlined above, our previous TTI studies have experimentally constructed various TTIs and NNIs using different ISIs within or between blocks, or with fixed interval ranges within a fixed-ISI stimulus sequence. In the current study, we utilized a more typical oddball task structure that has been used and published previously (McDonald et al., 2010) in order to test whether TTI and NNI effects could be observed when no manipulation was present. Thus, the distance (quantified by TTI and NNI) between matching stimuli can be considered the core determinant of component amplitudes because ISI and global probability are held constant, and a mixture of stimulus types are presented within each interval.

A novel regression approach (that has not been used previously to investigate TTI/NNI effects) explored the effect of intervals occurring within the stimulus sequence of this typical oddball task. This method was used to further explore the mechanism of TTI/NNI effects and determine whether unique variance in ERP components at various stages of information processing could be predicted by TTI/NNI. To facilitate exploration of differing stimulus-pathway specific effects, we aimed to examine the mechanism of interval effects within the separate processing streams. In addition, a fixed ISI was utilized to avoid any ISI/matching-stimulus interval confounds and the possibility of eliciting atypical ERP components.

In line with Steiner et al. (2014b), it was expected that increases in TTI and NNI would enhance N1 and P2 amplitudes, and we expected PN and SW to be unaffected by manipulations of interval. As the paradigm was a three-stimulus task with probabilities similar to those in Steiner et al. (2013b), we expected P300 components (P3a and P3b) to be affected only by TTI and not NNI. In addition, and in line with previous sequence and global probability studies (Sams et al., 1983; Polich, 1990b), we expected N2 amplitude to increase as matching-stimulus interval increased. Furthermore, based on Thomas et al.'s (2009) sequence study using a Go/NoGo task, we did not expect P1 to show a systematic increase as interval increased.

## Methods

### Participants

Participants were 28 undergraduate students from the University of Wollongong (*M*_age_ = 24.2, *SD* = 7.9 years; 18 females, all right handed), who completed this study as part of a research participation course requirement. Prior to the experiment, participants provided informed consent and were free to withdraw at any time without penalty. Those with self-reported neurological or psychiatric illnesses, and individuals taking psychotropic medication, were excluded. Participants self-reported that they had refrained from psychoactive substances for at least 12 h and from tea, coffee, alcohol, and cigarettes for at least 2 h prior to testing. All participants had normal or corrected-to-normal vision and self-reported normal hearing.

### Procedure

A demographic and screening questionnaire was completed by all participants before they were fitted with EEG recording apparatus. Prior to the experiment, participants completed an electrooculogram (EOG)/EEG calibration task (Croft and Barry, 2000). Participants were seated in an air-conditioned room 600–800 mm in front of a 48.3 cm (19″) Dell LCD monitor, and instructed to fixate on a 10 × 10 mm gray cross centered on a black background. Acoustic stimuli were delivered binaurally through Sony MDR V700 circumaural stereo headphones. Care was taken to ensure that fitting participants with headphones did not disturb electrode impedances or introduce unwanted artifact.

### Oddball paradigm

Participants completed a three-tone oddball task consisting of low probability targets (*p* = 0.10; 2000 Hz tone), rare nontargets (*p* = 0.10; 500 Hz tone), and a frequent standard (*p* = 0.80; 1000 Hz tone). All stimuli were 80 dB SPL, 336 ms duration (10 ms rise/fall; as per Cycowicz et al., 1996). A random stimulus order was fixed across subjects (identical to McDonald et al., 2010), with a total of 480 trials presented in a single block with a 1 s SOA. All participants were instructed to respond to target stimuli as quickly and as accurately as possible with a button press using their right hand on a Logitech® Precision game controller. Instruction was given to minimize movement as much as possible, but participants were not instructed to refrain from blinking (Verleger, 1991). The procedure was approved by the joint South Eastern Sydney/Illawarra Area Health Service and University of Wollongong Health and Medical Human Research Ethics Committee (Ethics Approval Number: HE11/185).

### Materials and apparatus

Continuous EEG data were recorded DC-70 Hz with a Neuroscan Synamps 2 digital signal-processing system and Neuroscan 4.3.1 Acquire software. Data were acquired from A2 and 30 scalp sites (Fp1, Fp2, F7, F3, Fz, F4, F8, FT7, FC3, FCz, FC4, FT8, T7, C3, Cz, C4, T8, TP7, CP3, CPz, CP4, TP8, P7, P3, Pz, P4, P8, O1, Oz, O2) with an electrode cap using tin electrodes. A1 was used as a reference and the cap was grounded by an electrode located midway between Fp1, Fp2 and Fz. Display and stimulus markers were controlled by a linked stimulus computer using Neurobehavioral Systems Inc. Presentation V 13.0 Build 01.23.09 software.

EOG was recorded using tin cup electrodes placed 2 cm above and below the left eye for vertical movements, and on the outer canthus of each eye for horizontal movements. Impedance was less than 5 kΩ for cap, EOG, and reference electrodes. Scalp and EOG potentials were amplified with a gain of 500 and digitally sampled at 1000 Hz.

### Data extraction and averaging procedure

Single trials containing omission (miss) or commission (false alarm) errors, or lengthy response times (>800 ms), were excluded. All participants responded accurately to at least 95% of trials (mean across-subject errors = 0.63%, *SD* = 1.0). EEG data were EOG corrected using the Revised Artifact-Aligned Average (RAAA) EOG Correction Program (Croft and Barry, 2000). Data were re-referenced to digitally linked earlobes and extracted offline using the Neuroscan Edit software, band-pass filtered (0.1–30 Hz, zero-phase shift, 24 dB/Octave), epoched for −100 ms pre- to 900 ms post-stimulus, and single trials were baselined to the prestimulus period. Data were manually inspected for any additional artifact; any contaminated single trials (< 4% of total trials) were excluded from analysis.

**Figure 1:**
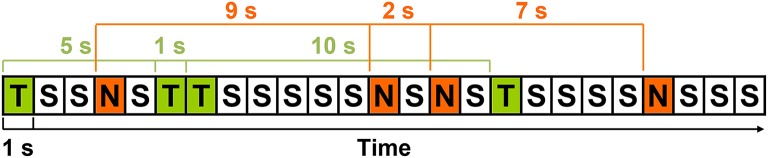
**An example of the stimulus sequence with targets (T), rare nontargets (N), and standards (S); each rectangle represents the 1 s SOA**. Three TTIs (5, 1, 10 s) and NNIs (9, 2, 7 s) are detailed in green and red, respectively.

The stimulus-to-matching-stimulus intervals separating the presentation of the 48 targets and 48 rare nontargets were calculated, yielding 47 intervals of each stimulus type (TTI range = 2–39 s; NNI range = 2–38 s). Separately for each condition, means were computed across-subjects for each interval-type (i.e., 2 conditions × 47 intervals), creating a total of 94 intervals. Artifact-contaminated single trials from individual subjects were not included in these across-subjects means. That is, 47 means per stimulus were computed, but not all 28 subjects contributed to each of these means (< 1% of trials). An example of the stimulus sequence and illustration of TTIs and NNIs is detailed in Figure 1. TTIs could include both rare nontargets and standards, and NNIs both targets and standards. For a similar quantification of high probability standards, an identical number of intervals were obtained by calculating the interval from the standard (S) preceding every second target (T) and rare nontarget (N; e.g., STS, SNS, STNS etc.). However, this produced a narrow interval range (standard-to-standard interval; SSI = 2–3 s), and subsequently, quantification of standard data was for topographic illustration and sequential processing analyses only; all analyses involving interval were restricted to targets and rare nontargets.

### Principal components analysis

The across-subjects averaged data from 30 scalp locations were submitted to a temporal Principal Components Analysis (PCA) using Dien's ERP PCA toolkit (v. 2.23; Dien, 2010) in MATLAB (The Mathworks, R14SP3). Following Dien (2012), an initial temporal PCA was conducted on the averaged data for all three conditions combined. However, this initial analysis failed to separate the data effectively, particularly after ~200 ms where broad differences in waveform morphology (temporally and spatially) are apparent. Hence, separate PCAs were conducted on the three conditions. Several PCAs were also conducted on epochs of different lengths; the epoch length entered into the final PCAs (−100 to 450 ms) was chosen because it separated the components most effectively^2^.

Data for each PCA were half-sampled to 275 time-points (variables) to reduce computation time and improve the cases/components ratio. Factors were quantified separately for each condition (1410 observations: 47 intervals × 30 sites). The PCA used the unstandardized covariance matrix with Kaiser normalization, and all 275 unrestricted factors underwent Varimax rotation, following Kayser and Tenke (2003). PCA factors were identified as ERP components and retained for analysis based on their latency, topography, and polarity of their conspicuous maximum loading. The microvolt-scaled factor scores (Dien, 2012) at the peak-latency for these components were output and entered into subsequent statistical analyses.

### Statistical analyses

To define component topography, regions within the sagittal and coronal planes from the nine core electrode sites (F3, Fz, F4, C3, Cz, C4, P3, Pz, P4) were assessed using within-subjects MANOVAs^3^. Topographic dimensions were the sagittal plane—frontal (F3, Fz, F4), central (C3, Cz, C4), parietal (P3, Pz, P4); and the coronal plane—left (F3, C3, P3), midline (Fz, Cz, Pz), and right (F4, C4, P4). Planned orthogonal contrasts for the sagittal plane compared frontal (F) and parietal (P) regions, and central (C) compared to the mean of frontal and parietal (F/P) regions. For the lateral plane, left (L) was compared to right (R), and the midline (M) to the mean of the left and right hemispheres (L/R). Once component distributions were identified, a cluster of electrode sites surrounding the region of maximal amplitude was selected; contour lines on topographic headmaps were also used as a guide.

The examination of interval effects in sequential processing involved a series of bivariate correlations, partial correlations, followed by multiple regression analyses, separately for targets and rare nontargets. First, correlations between intervals, amplitudes for each ERP component, and RTs (for targets only) were conducted; relationships between ERP components only were explored for standards. Partial correlations were subsequently used to separate interval and preceding-component contributions to these results for targets and rare nontargets. Next, separate stepwise multiple regressions, with each ERP component as the dependent variable, were conducted in order to examine the origin of interval effects in sequential processing for targets and rare nontargets. These included factors of interval and the amplitudes of all sequentially-preceding ERP components as predictors, with α = 0.05 as entry criteria. For example, for target P2 as a dependent variable, TTI, and previous target ERP components P1 and N1 would be entered as predictors. This stepwise analysis was also conducted for RT, with all target ERP components and TTI entered as predictors. Sequential processing was also explored in standards, however, due to the narrow interval range, only sequentially preceding ERP components were included as predictors. One-way tests were utilized for all analyzed predictions.

Two further items should also be noted. First, although ERP oddball research traditionally examines differences between stimulus types in order to confirm the manipulation check, this was not done here as we were interested in exploring only the shared variance between interval and ERP component amplitudes in stimulus-specific pathways. Second, as this paper details results for a number of dependent measures, the frequency of Type I errors increases. However, Howell (1997) argues that this increase in frequency of Type I errors cannot be controlled by adjusting α-levels, because the probability of Type I error remains the same. That is, testing one dependent variable has a 1 in 20 chance (5%) of a Type I error, and testing two dependent variables has a 2 in 40 (5%) chance of a Type I error.

## Results

### Grand means

**Figure 2:**
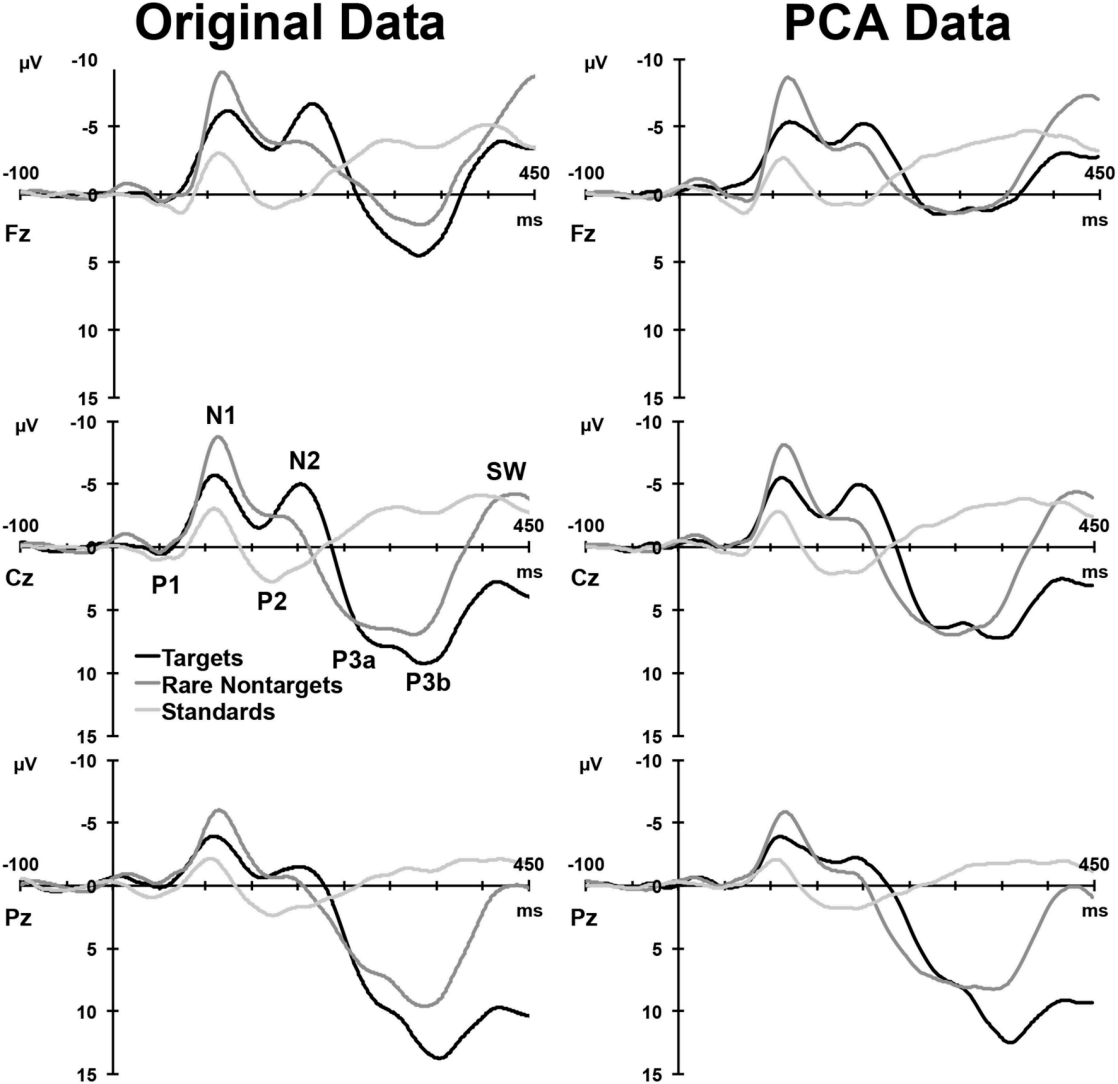
**Left:** Grand mean ERPs at midline sites for the three conditions. **Right**: ERP waveforms reconstituted from the components selected for analysis. A selection of clearly visible components in the raw waveforms is labeled at Cz.

**Figure 3:**
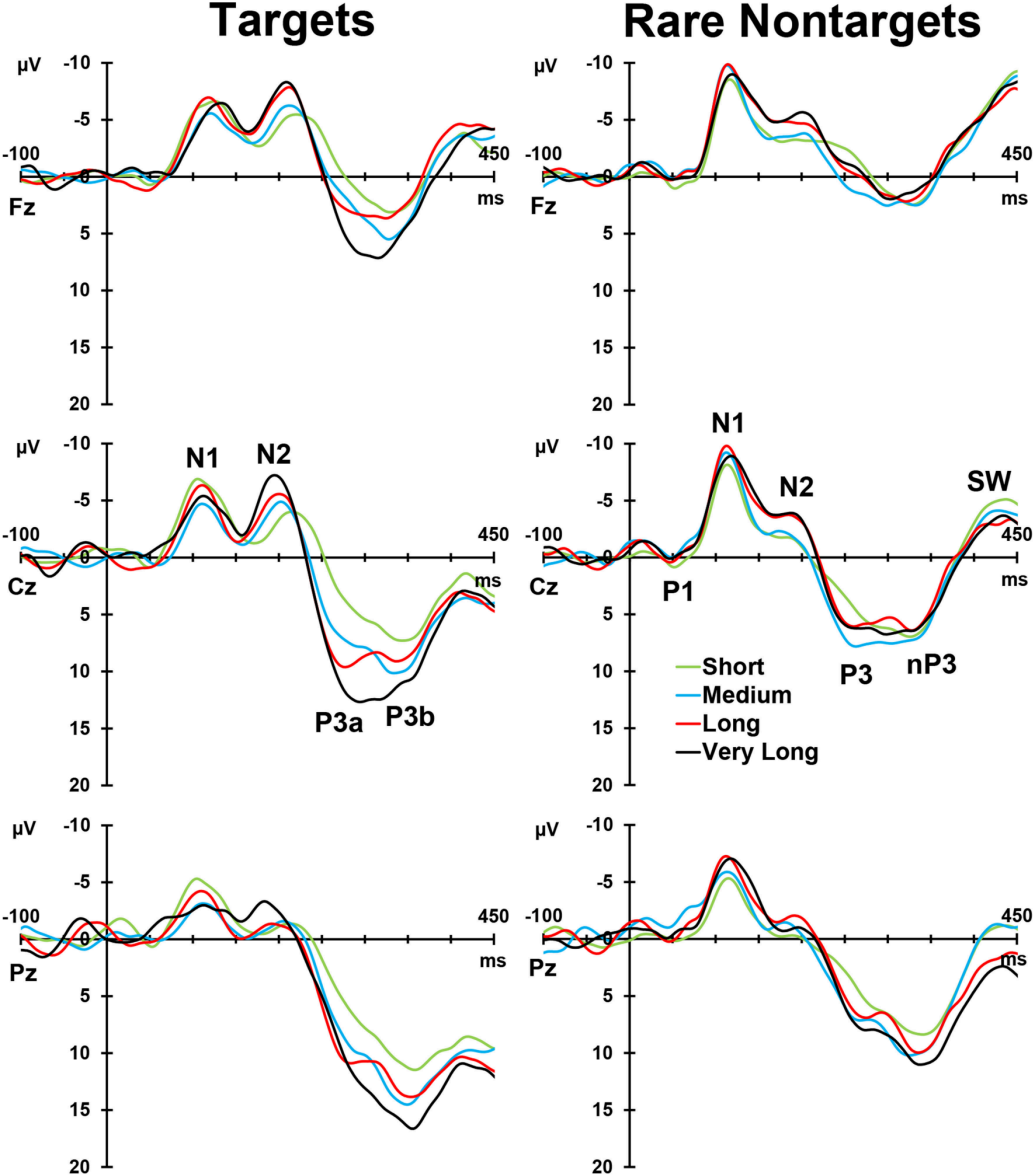
**Intervals sorted into short, medium, long, and very long intervals for targets (left) and rare nontargets (right) at midline sites**. Interval effects are apparent for target stimuli, particularly within the P300-latency range. Components visible in the raw waveforms are labeled at Cz for both stimulus types.

Figure 2, left column, depicts the grand mean ERPs for each of the conditions at midline sites. For illustrative purposes, intervals were sorted into short (2–4 s), medium (5–9 s), long (10–16 s), and very long intervals (17–39 s), and averages were formed separately for targets and rare nontargets; these means are shown in Figure 3. For targets (Figure 3, left column), clear TTI effects are visible at Cz in the negative deflection ~200 ms, and in the positive deflection ~300 ms. NNI effects are not as apparent for rare nontargets (Figure 3, right column), but there appears to be some evidence at ~200 ms at Fz and Cz.

### PCA outcomes

For each condition, all 275 factors were rotated. For targets, the first 5 factors accounted for 78.16% of the total variance, for rare nontargets, the first 6 accounted for 78.3% of the variance, and for standards, factors 1–6 explained 73.8% of the variance; these factors were extracted and retained for analysis. The sum of these extracted factors is illustrated in the right column of Figure 2. Although some small visual discrepancies are apparent when comparing the PCA data (right) to the original grand mean data (left), when correlated across the midline, the PCA and original data were highly similar for targets, *r*_(823)_ = 0.99, *p* < 0.001, rare nontargets, *r*_(823)_ = 0.99, *p* < 0.001, and standards, *r*_(823)_ = 0.98, *p* < 0.001.

**Figure 4:**
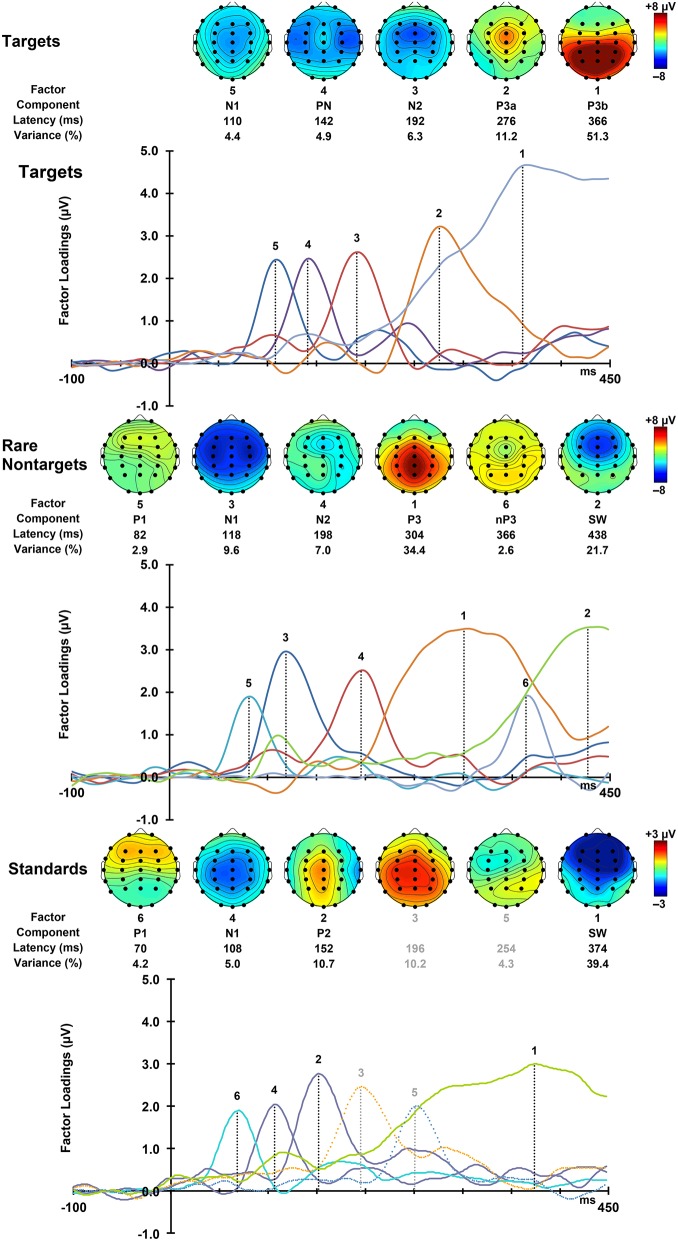
**Factor loadings for targets (top), rare nontargets (middle), and standards (bottom)**. Topographic plots for each extracted ERP component are illustrated above the factor loadings, together with factor rank, component label, latency, and variance explained, separately for each condition.

For each condition, the rescaled temporal factor loadings for each of the ERP components are displayed as a function of time in Figure 4. Topographic headmaps of the temporal components, averaged across subjects and intervals, are displayed above the factor loadings for each condition. The factor rank order, component label, latency, and percentage of variance explained for each of the rotated components are indicated below the headmaps. Components were labeled according to their latency, polarity, topographic distribution, and sequence. In latency order, for targets these were N1, PN, N2, P3a, and P3b; for rare nontargets: P1, N1, N2, P3, Novelty P3 (nP3), and SW; and for standards: P1, N1, P2, followed by unidentifiable components (a centro-parietal positivity and a frontally negative/parietally positive component; dashed lines, Figure 4 bottom panel, not analyzed), and SW. These other factors were not readily identifiable as ERP components, and each explained a small portion of the total variance (targets and standards < 4%, rare nontargets < 2.5%); consequently these were not considered for analysis and will not be discussed further.

### Component topographies

#### Targets

As shown in the top panel of Figure 4, target N1 was fronto-centrally negative (F > P: *F* = 5.23, *p* = 0.027, ${ŋ}_{p}^{2}$ = 0.10; C > F/P: *F* = 22.97, *p* < 0.001, ${ŋ}_{p}^{2}$ = 0.33), and was greatest at the midline (M > L/R: *F* = 39.79, *p* < 0.001, ${ŋ}_{p}^{2}$ = 0.46). This midline topography was greatest frontally and at the vertex (F > P × M > L/R: *F* = 33.76, *p* < 0.001, ${ŋ}_{p}^{2}$ = 0.42; C > F/P × M > L/R: *F* = 9.72, *p* = 0.003, ${ŋ}_{p}^{2}$ = 0.17). Consistent with this topography, N1 was measured as the mean across the central midline sites (FCz, Cz, CPz). These statistical effects are detailed in Table 1. To save space these will be omitted from the following text for the other components. Note that some of the pairs of greater than (>) and/or less than (<) signs are reversed from the text descriptions for the statistical entries in Table 1. This utilizes the logical equivalence of such pair reversals, facilitating the tabulation of component differences in each effect.

**Table 1:** **Analysis of topography for each ERP component and stimulus type**.

**Targets**	**N1**			PN^*^			**N2**			**P3a**			**P3b**					
**Effect**	***F***	***p***	**${ŋ}_{p}^{2}$**	***F***	***p***	**${ŋ}_{p}^{2}$**	***F***	***p***	**${ŋ}_{p}^{2}$**	***F***	***p***	**${ŋ}_{p}^{2}$**	***F***	***p***	**${ŋ}_{p}^{2}$**			
F > P	5.23	0.027	0.10							8.54	0.005	0.16	1177.10	**<0.001**	0.96			
C > F/P	22.97	<0.001	0.33	74.68	<0.001	0.62	39.88	<0.001	0.46	53.32	<0.001	0.54	8.04	0.007	0.15			
L < R										5.52	0.023	0.11						
M > L/R	39.79	**<0.001**	0.46				11.47	0.001	0.20	133.25	**<0.001**	0.74	54.00	<0.001	0.54			
F > P × L < R													39.08	<0.001	0.46			
F > P × M > L/R	33.76	<0.001	0.42	15.44	<0.001	0.25	46.21	**<0.001**	0.50				173.27	**<0.001**	0.79			
C > F/P × L < R										14.77	<0.001	0.24	57.34	<0.001	0.55			
C > F/P × M > L/R	9.72	0.003	0.17	182.02	**<0.001**	0.80				97.47	<0.001	0.68	47.97	<0.001	0.51			
**Rare nontargets**	**P1**			**N1**			**N2**			**P3**			**nP3**			**SW**		
**Effect**	***F***	***p***	${ŋ}_{p}^{2}$	***F***	***p***	${ŋ}_{p}^{2}$	***F***	***p***	${ŋ}_{p}^{2}$	***F***	***p***	${ŋ}_{p}^{2}$	***F***	***p***	${ŋ}_{p}^{2}$	***F***	***p***	${ŋ}_{p}^{2}$
F > P	16.61	**<0.001**	0.27	32.96	**<0.001**	0.42				187.25	<0.001	0.80	8.82	**0.005**	0.16	**186.60**	**<0.001**	**0.80**
C > F/P				100.08	<0.001	0.69	4.44	0.041	0.09	241.29	<0.001	0.84	18.39	<0.001	0.29	25.50	<0.001	0.36
L < R	4.78	0.034	0.09				24.55	<0.001	0.35							7.01	0.011	0.13
M > L/R										448.00	**<0.001**	0.91	22.92	<0.001	0.33	61.82	<0.001	0.57
F > P × L < R							7.20	0.010	0.14									
F > P × M > L/R				13.19	0.001	0.22	94.45	**<0.001**	0.67	21.85	<0.001	0.32	8.64	0.005	0.16	5.27	0.026	0.10
C > F/P × L < R	21.32	<0.001	0.32	8.95	0.004	0.16	20.75	<0.001	0.31				27.09	<0.001	0.37			
C > F/P × M > L/R	5.34	0.025	0.10	193.67	**<0.001**	0.81				31.82	<0.001	0.41	93.52	<0.001	0.67	28.55	<0.001	0.38
**Standards**	**P1**			**N1**			**P2**			**SW**								
**Effect**	***F***	***p***	${ŋ}_{p}^{2}$	***F***	***p***	${ŋ}_{p}^{2}$	***F***	***p***	${ŋ}_{p}^{2}$	***F***	***p***	${ŋ}_{p}^{2}$						
F > P	37.73	**<0.001**	0.45				4.33	0.043	0.09	112.49	**<0.001**	0.71						
C > F/P				24.81	**<0.001**	0.35	7.25	0.010	0.14	9.91	0.003	0.18						
L < R							25.15	<0.001	0.35	8.54	0.005	0.16						
M > L/R				17.27	<0.001	0.27	119.13	**<0.001**	0.72	20.15	<0.001	0.30						
F > P × L < R				4.94	0.031	0.10				13.52	0.001	0.23						
F > P × M > L/R										31.45	<0.001	0.41						
C > F/P × L < R				10.27	0.002	0.18	38.74	<0.001	0.46	53.76	<0.001	0.54						
C > F/P × M > L/R							46.97	<0.001	0.51									

*For each component, paired effect and statistic underlining indicates a reversal of that effect. Reversals of any two directional indicators within an effect indicate a statistically equivalent interpretation. Bolded p-values indicate dominant topography used as a guide to select sites analyzed. F, frontal; P, parietal; C, central; L, left; R, right; M, midline; < and > indicate direction of effect; and × denotes interactions. All F-tests are reported with (1, 46) degrees of freedom*.

* *Analysis includes temporal sites*.

PN was largest at temporal sites, with a midline reduction that was largest frontally and at the vertex. In line with this, temporal sites close to the center were selected for further analysis (T7, C3, C4, T8; see Figure 4, top panel). N2 had larger amplitudes centrally and in the midline, particularly at the front. Consistent with this fronto-central topography, analyzed sites included FC3, FCz, and FC4. P3a was fronto-central, and largest at the midline and vertex. There was also some right hemispheric enhancement that was greatest centrally. Thus, fronto-midline sites were selected for analysis (FCz, Cz). P3b was maximal parietally, with enhancements centrally and in the midline that were both larger on the right. These interacted to produce a vertex enhancement. As such, parietal-midline-central sites were selected for analysis (CPz, P3, Pz, P4).

#### Rare nontargets

P1 to rare nontargets was frontally distributed, with a slight enhancement in the right that was greatest centrally; amplitudes were also reduced at the vertex. A mean across electrode sites within the frontal region (F3, Fz, F4) was taken to define P1. N1 had a strong fronto-central topography (as such, a mean across F3, FC3, C3, F4, FC4, and C4 was used for subsequent analyses), which was reduced in the midline, and was slightly elevated centrally on the left. N2 was greatest at fronto-midline sites (mean across Fz and FCz was used to define topography), with enhancements centrally and in the right hemisphere; the right enhancement was greatest parieto-centrally. There was a slight reduction at central sites compared to the mean of frontal and parietal sites. The P3 component had a strong centro-parietal and midline distribution. A midline enhancement was greatest parietally, and amplitudes were also elevated at the vertex. In line with this topographic distribution, a mean across midline parieto-central sites (Cz, CPz, Pz) was computed for subsequent analyses. The nP3 component was dominant parietally (thus, P3, Pz, and P4 were used to define topography), and reduced centrally and in the midline. These interacted to produce a vertex minimum. The parietal enhancement was greatest in the midline, and the central reduction was greatest in the left hemisphere. SW was largest at frontal and midline sites (Fz and FCz were used to compute mean amplitudes for SW); these interacted, and frontal sites were greatest at the midline. Amplitudes were also enhanced centrally and on the right.

#### Standards

For standards, P1 was largest frontally, thus a mean across frontal electrodes (F3, Fz, F4) was used for subsequent analyses. N1 was greatest centrally and in the midline (FCz, Cz, and CPz were used to define topography), with larger amplitudes in the left hemisphere, both parietally and centrally. P2 was largest at the midline (thus a mean across FCz, Cz, and CPz was taken); there were also enhancements parietally, centrally, and on the left. Centrally, amplitudes were largest on the left and at the vertex. SW had a strong fronto-midline topography (Fz and FCZ were used to compute the mean), with larger amplitudes centrally, and on the right. Frontal amplitudes were greatest on the right, parietal amplitudes were largest in the midline, and the right enhancement was largest centrally.

### Correlations and regression

#### Targets

Table 2 illustrates the bivariate correlations between intervals and ERP components for each stimulus condition. TTI was positively correlated with target N1, P3a, P3b, and RT, and inversely correlated with N2. N1 was inversely correlated with N2 and positively correlated with P3a amplitudes. RT was positively correlated with PN and negatively correlated with SW.

**Table 2:** **Bivariate correlations between intervals and proceeding ERP components for each condition**.

**Targets**	**TTI**	**N1**	**PN**	**N2**	**P3a**	**P3b**	**RT**
TTI	1.000						
N1	0.253^*^	1.000					
PN	−0.092	−0.002	1.000				
N2	−0.443^**^	−0.373^**^	−0.051	1.000			
P3a	0.452^**^	0.359^**^	−0.071	−0.110	1.000		
P3b	0.284^*^	0.117	−0.238	−0.179	−0.081	1.000	
RT	0.275^*^	−0.080	0.361^**^	−0.106	0.002	−0.288^*^	1.000
**Rare Nontargets**	**NNI**	**P1**	**N1**	**N2**	**P3**	**nP3**	**SW**
NNI	1.000						
P1	0.067	1.000					
N1	−0.141	0.408^**^	1.000				
N2	−0.295^*^	−0.114	−0.032	1.000			
P3	0.100	0.217	0.008	−0.270^*^	1.000		
nP3	−0.106	0.104	0.154	−0.114	0.037	1.000	
SW	0.168	−0.085	−0.400^**^	−0.219	−0.026	0.008	1.000
**Standards**	**P1**	**N1**	**P2**	**SW**			
P1	1.000						
N1	−0.114	1.000					
P2	−0.102	−0.257^*^	1.000				
SW	−0.123	−0.094	−0.245^*^	1.000			

*df = 45*,

* *p < 0.05*,

** *p < 0.01*.

Partial correlations were also computed to test for shared variance between TTI and amplitudes for each ERP component, whilst controlling for variance contributed by amplitudes from each preceding ERP component. Table 3 shows that the relationship between TTI and N2, P3a, P3b, and RT all remain constant after controlling for sequentially preceding ERP component amplitudes.

**Table 3:** **Partial correlations between matching-stimulus intervals and amplitudes for each ERP component (separately for TTI and NNI), controlling for variance contributed by sequentially preceding ERP component amplitudes**.

**Targets**	**PN**	**N2**	**P3a**	**P3b**	**RT**
TTI	−0.094 (44)	−0.396^**^ (43)	0.441^**^ (42)	0.307^*^ (41)	0.457^**^ (40)
**Rare Nontargets**	**N1**	**N2b**	**P3**	**nP3**	**SW**
NNI	−0.185 (44)	−0.292^*^ (43)	−0.001 (42)	−0.130 (41)	0.050 (40)

*r (df)*,

* *p < 0.05*,

** *p < 0.01*.

In the stepwise multiple regression model, TTI did not explain a significant portion of the variance in N1, and PN was not predicted by N1 and/or TTI. With PN excluded from the model (i.e., it was not a significant predictor), TTI (β = −0.373) and N1 (β = −0.279) explained 27% of the variance in N2, *F*_(1, 46)_ = 8.11, *p* = 0.001. TTI (β = 0.452) accounted for 21% of the variance in P3a, *F*_(1, 46)_ = 11.57, *p* = 0.001, after N1, PN, and N2 were excluded from the model. TTI (β = 0.309) accounted for a significant amount (10%) of the total variance in P3b, *F*_(1, 46)_ = 4.74, *p* = 0.035. RT was predicted by PN (β = 0.321), P3b (β = −0.324), and TTI (β = 0.396), *F*_(1, 46)_ = 6.68, *p* = 0.001, which accounted for 32% of overall RT variance.

#### Rare nontargets

Bivariate correlations showed that NNI was negatively correlated with N2 amplitudes (Table 2), rare nontarget P1 was directly related to N1, N1 was inversely correlated with SW, and N2 was inversely correlated with P3. A partial correlation (Table 3) showed that the negative relationship between NNI and N2 amplitudes remained after controlling for variance contributed by P1 and N1.

Stepwise multiple regression showed that NNI did not contribute to a significant proportion of the variance in P1. After NNI was excluded from the model, P1 (β = 0.408) explained 17% of the variance in N1, *F*_(1, 46)_ = 9.00, *p* = 0.004. N2 was significantly predicted by NNI (β = −0.295) after P1 and N1 were excluded from the model, *F*_(1, 46)_ = 4.30, *p* = 0.044, accounting for 9% of the variance. P3 and nP3 were not predicted by preceding ERP components or NNI. N1 (β = −0.400) accounted for 16% of the variance in SW, *F*_(1, 46)_ = 8.57, *p* = 0.005.

#### Standards

As illustrated in the lower section of Table 2, bivariate correlations showed that there was a weak inverse relationship between N1 and P2, and between P2 and SW. In the stepwise multiple regression model, none of the ERP components elicited by standards were predicted by sequentially preceding components.

## Discussion

We examined the determinants of the matching-stimulus interval effect in components of the ERP using a conventional three-tone oddball task. Findings were broadly compatible with our previous investigation (Steiner et al., 2014b). Multiple regression showed that for targets, N1 and TTI predicted N2, TTI predicted P3a and P3b, and PN, P3b, and TTI predicted RT. For rare nontargets, P1 predicted N1, NNI predicted N2, and N1 predicted SW. In addition, target N1 and PN, and rare nontarget P1, P3, and nP3, and all standard components, were not significantly predicted by the variables examined here. Results from the stepwise multiple regressions and correlations were largely comparable. Findings are suggestive of a temporal mechanism that affects specific stages of sequential processing.

### Correlation and regression findings

Bivariate correlations showed that TTI was directly correlated with P3a, P3b, and RT, and inversely correlated with N2. These relationships remained constant after controlling for sequentially preceding ERP component amplitudes. Larger N1 amplitudes were associated with smaller N2 and larger P3a amplitudes, and larger PN amplitudes were linked with faster RTs. Two further relationships were detected with correlations, but did not reach significance in the stepwise multiple regression models: longer TTIs were associated with smaller N1 component amplitudes, and larger SW amplitudes were associated with slower RTs.

Multiple regression analyses showed that for targets, N1 and TTI explained a significant proportion of the unique variance in N2, TTI predicted P3a and P3b, and PN, P3b, and TTI predicted RT. Together, these results show that increases in TTI are associated with larger N2, P3a and P3b amplitudes to targets, smaller N1 amplitudes and longer RTs.

Correlations showed an inverse relationship between NNI and N2 (which remained after controlling for variance contributed by P1 and N1 amplitudes), a direct relationship between P1 and N1, and a negative correlation between N1 and SW. Larger N1 amplitudes were associated with smaller P1 and larger SW amplitudes, and larger N2 amplitudes were related to larger P3 component amplitudes. N2 was also found to be inversely correlated with rare nontarget P3, a finding that was not apparent in the stepwise multiple regression.

In the multiple regression model for rare nontargets, P1 explained unique variance in N1, NNI predicted N2, and N1 determined SW. None of the predictors examined explained a significant proportion of the unique variance in target N1 and PN amplitudes, or rare nontarget P1, P3, and nP3^4^. This indicates that in this paradigm, NNI modulates rare nontarget N2 component amplitudes only.

For standard ERP component amplitudes, bivariate correlations revealed a weak negative correlation between N1 and P2, and P2 and SW indicating that larger P2 amplitudes were associated with larger N1 and SW amplitudes. Multiple regression showed that component amplitudes to standards did not have a significant proportion of variance explained by any of the preceding ERP components.

### Comparison with previous work

Despite large differences in paradigms (fixed vs. variable ISI, three vs. two stimuli, and low- vs. equal-probability targets and nontargets), there was some overlap between the current findings and Steiner et al. (2014b): both studies found that P3b was determined by TTI, and PN and SW were not determined by matching-stimulus intervals. Due to the above-mentioned task discrepancies, different components were elicited in the two studies, making some direct comparisons impossible. For instance, in our previous investigation a clear P2 was obtained, but as outlined above, this was absent to targets and rare nontargets in the current study (most likely due to the overlapping N2). On the other hand, P1, N2, P3a, and nP3 were extracted in the present study, but were not identified in Steiner et al. (2014b). Furthermore, and in line with local and global probability studies (Sams et al., 1983; Polich, 1990b), target and rare nontarget N2 was found to increase with longer TTIs and NNIs, and nontarget P1 was not affected by NNI (Thomas et al., 2009).

Unlike Steiner et al. (2014b), here, N1 amplitude was inversely related to TTI, which may be due to contributions from a resolving contingent negative variation (CNV). Specifically, the regularity in the current paradigm (fixed ISI) may have led to a stronger CNV (cf. Steiner et al., 2014b), which has been shown to influence the N1 (Karamacoska et al., 2015). Future research could clarify this by employing the same paradigm used here, but varying the SOA around a 1 s mean to obviate the CNV and test whether the N1 enhancement to increases in TTI observed in Steiner et al. (2014b) can be replicated in this paradigm.

The regression analyses produced a number of other novel findings. For targets, RT was predicted by PN, P3b, and TTI, a highly novel finding that is congruent with the functional roles of PN and P3b in sequential processing in active tasks (i.e., PN is thought to be the attentional trace of a stimulus, facilitating rapid selection of task relevant information, (Näätänen, 1990); and P3b indexes a monitoring process that is associated with both stimulus- and response-related processing; Verleger et al., 2005). For rare nontargets, N1 was predicted by P1, a similar relationship to reports from P50 paired-click paradigms, where the N1 following the second P50 is often reduced (Hanlon et al., 2005). Rare nontarget SW was predicted by N1, an unexpected finding that is difficult to interpret, but may reflect a link between the sensory memory trace of a stimulus (Näätänen, 1990), and decision-related processing time (Ruchkin et al., 1988); this is speculative and requires replication in future research.

The current data and Steiner et al. (2014b) indicate that interval effects are present in the very different processing stages reflected in N1, P2, N2, P3a, and P3b, suggestive of activity within separable stimulus-processing pathways related to attention, memory maintenance, and the monitoring of task-related requirements (decision-making and responding). These are broad aspects of executive control, and differences in NNI findings (e.g., Steiner et al., 2013b vs. Steiner et al., 2014a) suggest that they are largely influenced by the attentional set (Sawaki and Katayama, 2006). The current data also indicate a divergence of stimulus processing after N2, with no NNI effects apparent in subsequent rare nontarget components, suggesting that matching-stimulus timing may not affect the involuntary shift in attentional processing of irrelevant stimuli indexed by nontarget P3 and nP3. Further, the absence of interval effects in PN suggests that stimulus-timing may not be relevant for the process underlying this component.

Intracranial and dipole localization studies suggest that sources of the components sensitive to interval effects include the supra-temporal plane of the primary auditory cortex (N1; Vaughan and Ritter, 1970), auditory cortex and the reticular activating system (P2; Ross and Tremblay, 2009), frontal cortex (N2; Giard et al., 1990), hippocampal-thalamo-cortical network (P3a; Klostermann et al., 2006; Polich, 2007), and temporo-parietal cortex (P3b; Verleger, 2008). Rather than these specific component generators each showing differential and separate sensitivities to TTIs/NNIs, it may be that the interval effects we observed are the result of activity within a diffuse system that connects sensory and perceptual registries with cognitive mechanisms (e.g., working memory).

Interval effects have been adequately demonstrated in auditory and visual modalities. Future work should extend these findings into tactile and pain modalities. For example, Tanaka et al. (2008) investigated the effects of ISI and modality (auditory, tactile, visual, and pain) on source activation to elucidate the temporal sequence of multimodal and modality-specific activations. Interestingly, for the auditory modality (as used here), Tanaka et al. (2008) reported greater activation with longer ISIs in the superior-temporal gyrus, which may be the result of a refractory period effect (as reported in Budd et al., 1998). Tanaka et al. (2008) also reported a common temporal sequence of activation across the four sensory modalities (early then late sensory cortices, anterior cingulate, then hippocampus). Future research could map TTI and NNI effects to a well-established sequential processing schema, such as the multimodal schema detailed in Tanaka et al. (2008), or the equiprobable Go/NoGo processing schema proposed in Barry et al. (Barry and De Blasio, 2013, 2015; Borchard et al., 2015; Barry et al., 2016). Such an investigation would elucidate the locus of interval effects in sequential processing, and add value to interpretations of its role in cognitive processing.

We specifically adopted a regression approach to examine the contribution of both matching-stimulus intervals and sequentially preceding ERP components to the full range of components elicited by the different stimulus categories. It should be noted that other work examining trial-by-trial changes in ERP component amplitudes (e.g., Mars et al., 2008; Kolossa et al., 2013) have focused on the probability at each stimulus presentation (and the P3 only), which, whilst being an important question to address, was not the focus here. It should be noted, that in our study, the preceding stimulus for each TTI and NNI trial was always a standard stimulus, with the exception of one trial for each stimulus type, making comparison between our approach and others exploring trial-by-trial fluctuations in ERP component amplitudes dubious.

It was raised in the review process that headphones may generate artifactual contamination of the EEG. We are not aware of published data on this issue, but colleagues who have observed such effects have traced them to faulty signal generators. We have seen no evidence of such artifact in the hundreds of EEG and ERP studies from our laboratory, and think it unlikely to impact the present ERPs. Also, our stimuli here are ramped with 10 ms rise/fall time to reduce auditory transients, and this, coupled with the small signal voltages involved, serves to further reduce possible electromagnetic field transients.

### Conclusions

This study aimed to replicate Steiner et al. (2014b), increase the generalisability of previous work, and further explore the locus of matching-stimulus interval effects in sequential processing. In our previous investigation, we hypothesized that interval effects may represent a global refractory period effect progressing throughout the sequential processing stages reflected in the ERP components. Together, data indicate that changes in TTI/NNI affect non-sequential aspects of stimulus processing. This is suggestive of parallel-processing pathways, some of which are unaffected by TTI/NNI, arguing against a *global* stimulus-pathway recovery cycle mechanism. However, interval effects may be the result of very long component generator recovery cycles, or alternatively, activation within *particular* streams of stimulus processing, such as those involved in attention and working memory.

Viewed as a whole, findings may suggest at least two different processes. One possible explanation is that interval effects are associated with activation within stimulus-specific processing pathways, such as a memory-updating processes. Alternatively, interval effects might represent very long recovery cycles, affecting each of the component generators sensitive to TTI/NNI manipulations. For instance, Barry et al. (2011) reported ERP component amplitudes up to 50 μV in a long ISI study (50–70 s), suggesting that component magnitudes may continue to increase well beyond the ranges reported in typical ERP-style short-ISI studies (compared to autonomic-style long-ISI studies). Future research should seek to replicate these findings, and further explore a broader range of interval effects (e.g., from 1 s to 2 min) in a variety of tasks known to elicit ERP components different to those already examined.

In sum, this study applied a novel approach to examine the origin of interval effects in sequential processing. We used multiple regression to explore changes in component magnitude to a wide range of intervals occurring within the stimulus sequence of a typical auditory three-stimulus oddball task. Findings were broadly compatible with our previous investigation: TTI explained a significant proportion of the unique variance in N2, P3a, and P3b, and NNI explained unique variance in rare nontarget N2. In addition, target PN, and rare nontarget P1, P3, and nP3, and standard components were not significantly determined by any of the predictors examined. Together, this suggests that the matching-stimulus interval effect is underpinned by a mechanism that affects several stimulus-processing chains associated with attention, memory updating, and decision/response monitoring. This argues against a global refractory period mechanism, but may indicate either very long component generator recovery cycles, or increased activation within each particular pathway.

The persistence of matching-stimulus interval effects regardless of the stimulus context highlights the importance of the timing between repeated events in determining ERP component amplitudes. Future work should take these results into account when designing similar tasks.

## Author contributions

GS designed and carried out the study, collected the data, conducted all analyses and wrote the manuscript draft. RB assisted with the study design, supervised the process, and both RB and CG critically reviewed the drafted manuscript.

### Conflict of interest statement

The authors declare that the research was conducted in the absence of any commercial or financial relationships that could be construed as a potential conflict of interest.

## Footnotes

^1^However, effects of stimulus sequence, global probability, and ISI have not been widely explored in P1.

^2^We were also concerned about possible latency variability attributable to the large differences in interval length (>35 s). Thus, additional PCAs that included only the shortest (2–4 s) and longest (17–39 s) intervals were conducted separately on targets and rare nontargets. These analyses both produced components identical to the final PCAs conducted on the entire interval series.

^3^Except for Processing Negativity (PN), where central electrode pairs F3/4, C3/4, and P3/4 were replaced with corresponding outer electrode pairs F7/8, T7/8, and P7/8, for more effective analysis of this temporally-distributed component.

^4^It should be noted that the raw ERP waveforms (Figure 3, right column) suggest that the broad P3-deflection at Pz is influenced by NNI. Temporal PCA may have selected a subset of variance shared by multiple P3 components, some of which may not be sensitive to interval, resulting in the absence of an NNI effect.
